# Contrasting amino acid profiles among permissive and non-permissive hosts of *Candidatus* Liberibacter asiaticus, putative causal agent of Huanglongbing

**DOI:** 10.1371/journal.pone.0187921

**Published:** 2017-12-13

**Authors:** Mamoudou Sétamou, Olufemi J. Alabi, Catherine R. Simpson, John L. Jifon

**Affiliations:** 1 Texas A&M University-Kingsville Citrus Center, Weslaco, United States of America; 2 Department of Plant Pathology & Microbiology, Texas A&M AgriLife Research and Extension Center, Weslaco, TX, United States of America; 3 Department of Horticultural Sciences, Texas A&M AgriLife Research and Extension Center, Weslaco, TX, United States of America; USDA Agricultural Research Service, UNITED STATES

## Abstract

Huanglongbing is a devastating disease of citrus. In this study, a comprehensive profile of phloem sap amino acids (AA) in four permissive host plants of *Candidatus* Liberibacter asiaticus (*C*Las) and three non-permissive Rutaceae plants was conducted to gain a better understanding of host factors that may promote or suppress the bacterium. The AA profiles of *Diaphorina citri* nymphs and adults were similarly analyzed. A total of 38 unique AAs were detected in phloem sap of the various plants and *D*. *citri* samples, with phloem sap of young shoots containing more AAs and at higher concentrations than their mature counterparts. All AAs detected in phloem sap of non-permissive plants were also present in *C*Las -permissive hosts plus additional AAs in the latter class of plants. However, the relative composition of 18 commonly shared AAs varied between *C*Las -permissive hosts and non-permissive plants. Multivariate analysis with a partial least square discriminant methodology revealed a total of 12 AAs as major factors affecting *C*Las host status, of which seven were positively related to *C*Las tolerance/resistance and five positively associated with *C*Las susceptibility. Most of the AAs positively associated with *C*Las susceptibility were predominantly of the glutamate family, notably stressed-induced AAs such as arginine, GABA and proline. In contrast, AAs positively correlated with *C*Las tolerance/resistance were mainly of the serine family. Further analysis revealed that whereas the relative proportions of AAs positively associated with *C*Las susceptibility did not vary with host developmental stages, those associated with *C*Las tolerance/resistance increased with flush shoot maturity. Significantly, the proline-to-glycine ratio was determined to be an important discriminating factor for *C*Las permissivity with higher values characteristic of *C*Las -permissive hosts. This ratio could be exploited as a biomarker in HLB-resistance breeding programs.

## Introduction

Huanglongbing (HLB; citrus greening) disease has long been a serious disease of citrus in Asia [1]. In recent years, the disease has spread throughout major citrus growing areas in the Americas. The substantial economic damage caused by HLB to the Florida citrus industry is well documented [2], and the disease is threatening the sustainability of citrus production in other major citrus producing states such as California and Texas. Considerable efforts and resources are being expended to control the spread of HLB in the Americas using a three-pronged approach of propagation of clean nursery stock, area-wide vector control and rogueing of infected trees [3].

The most prevalent of the three fastidious, phloem-inhabiting putative alpha-proteobacterial agents of HLB in the U.S. is *Ca*. Liberibater asiaticus (*C*Las). CLas is spread by the oligophagous Asian citrus psyllid *Diaphorina citri* Kuwayama, 1908 (Hemiptera: Liviidae) that feeds and develops exclusively on plants within the Rutaceae family including *Citrus* spp. and *Murraya* spp. Both *D*. *citri* nymphs and adults can acquire and transmit *C*Las Although many *Citrus* (sweet orange, grapefruit, limes, lemons, etc.) and non-*Citrus* (curry leaf, orange jasmine, etc.) rutaceous species are suitable for *D*. *citri* reproduction and development [4–6], the performance of *C*Las varies among Rutaceae plants [7]. Most commercially grown citrus species are permissive hosts, allowing for *C*Las multiplication and HLB development [1]. In contrast, curry leaf (*Murraya koenigii*) and orange jasmine (*Murraya exotica*) are non-permissive to *C*Las and thus HLB tolerant/resistant [7]. Some non-*Citrus* rutaceous plants such as white sapote (*Casimiroa edulis*) are neither hosts to *D*. *citri* [6] nor permissive to CLas [7]. Furthermore, the Madagascar periwinkle (*Catharanthus roseus* (L.) G.Don (Gentianales: Apocynacae), supports *C*Las replication and growth [8] even though it is a non-host of *D*. *citri*. *C*Las also readily multiplies within the hemolymph and body of *D*. *citri* [9–11], indicating further complexity of the HLB pathosystem. Based on observed differential *C*Las responses reported in numerous [1,7,12] studies, it is reasonable to postulate that phloem saps of *C*Las -permissive hosts (i.e. *Citrus* spp. and periwinkle) as well as *D*. *citri* hemolymph and tissue contain unique growth factors that facilitate *C*Las replication. Contrariwise, phloem saps of non-permissive plants (i.e. *Murraya* spp.) may contain *C*Las -suppressing factors. Since *C*Las is a yet-to-be-cultured fastidious bacterium [1], it is important to gain a better understanding of potential factors regulating its growth and replication.

Most bacteria have limited metabolic capabilities and mainly depend on their hosts for energy and growth substrates [13]. The fastidious nature of *C*Las suggests that it likely relies on its hosts to meet its nutritional needs. Indeed, an analysis of the complete *C*Las genome indicates that it has a limited ability for aerobic respiration and is likely auxotrophic for at least five amino acids [14]. *C*Las infection was also reported to differentially affect the expression of heat shock proteins in *D*. *citri* adults and nymphs [15]. Furthermore, the observed down-regulation of hexamerin, an amino acid storage protein in insects [16] led Vyas et al. [15] to suggest that *C*Las may modulate free amino acid (FAA) availability in its host through regulation of expression of amino acid (AA) storage protein genes. Taken together, amino acids appear to play a significant role in host- *C*Las interactions and they may be required for the bacterium host nutritional exploitation, growth and transmission processes.

The importance of young expanding citrus flush shoots for *C*Las acquisition [10,17] and transmission [18,19] by *D*. *citri* has been well documented. It has also been shown that the phloem sap of young and expanding citrus flush shoots contain significantly greater numbers and concentrations of individual amino acids than mature shoots [20]. While the amino acid composition of citrus phloem sap [20,21], periwinkle [12], psyllid hemolymph [22] and the non-citrus Rutaceae orange jasmine and curry leaf [23] have been documented, it is unclear how the AA profiles of *C*Las -permissive hosts and non-permissive Rutaceae plants compare to each other.

To address these knowledge gaps, the goal of this study was to perform a comparative analysis of amino acid profiles of *C*Las -permissive hosts and non-permissive Rutaceae plants to identify key amino acids that may act as *C*Las growth promoting and/or suppressing factors. Such *C*Las -promoting, host-encoded amino acids may be utilized for *in vitro* culturing of the bacterium or exploited for HLB management. In addition, putative discriminating AAs could be harnessed as biomarkers for quick screen of candidate *C*Las tolerant and/or susceptible *Citrus* spp. in breeding programs.

## Materials and methods

### Insects

*Diaphorina citri* adults and nymphs were obtained from a laboratory-reared colony at the Texas A&M University-Kingsville Citrus Center, Weslaco, Texas. The colony was established using psyllid adults collected from a mature grapefruit block in 2006, prior to the first detection of HLB in Texas [24]. Routine cultures of the psyllid were maintained on a mixture of caged orange jasmine (*Murraya exotica* L.), grapefruit (*Citrus* × *paradisi* Metcalfd.) and sweet orange (*C*. × *sinensis* (L.) Osbeck.) plants at 25±5°C with 14 h:10 h L:D cycle and 65±5% RH. No addition of feral psyllids was made to the colony since 2006 and regular PCR testing (twice a year) has established the colony as *C*Las -negative.

### Plants

The analyses were conducted on three commonly grown citrus species and known hosts of the ACP and *C*Las, two ornamental CLas tolerant/resistant Rutaceae hosts of *D*. *citri*, a *C*Las tolerant/resistant and psyllid non-host Rutaceae plant, and a *C*Las -permissive non-Rutaceae psyllid non-host plant (Table 1). All experimental plants were grown in 7.6 L pots containing a commercial potting mix (Metro-Mix #2; Sun Gro Horticulture Inc., Agawam, MA) in a greenhouse at Texas A&M University Kingsville Citrus Center facility. All citrus host plants were grafted onto sour orange (*Citrus × aurantium* L.) rootstock and were ca. 2 year-old at the time of the experiment. Own-rooted curry leaf, orange jasmine and periwinkle plants (12-24-month-old) were purchased from a certified local nursery in McAllen, Texas, then transferred into the 7.6 L rearing pots. All experimental plants were determined to be *C*Las -negative based on standard qPCR tests [25], then grown for 6 months under similar conditions in the greenhouse (25–40°C) between March and September 2014 prior to phloem sap collection. The plants were watered daily or as needed to prevent any hydric stress, uniformly fertilized with a complete fertilizer (Peters Professional 20-20-20 General Purpose; The Scotts Company, Marysville, OH) at the rate of 5 g/pot monthly, and sprayed as needed with imidacloprid (Admire Pro) or spirotetramat (Movento) (Bayer CropScience, Research Triangle Park, NC) for insect and mite control.

**Table 1:** List of plant species evaluated in this study and their permissiveness to *Candidatus* Liberibacter asiaticus (*C*Las) or the Asian citrus psyllid (ACP).

Common name	Botanical name	Cultivar	CLas status^a^	ACP status^b^
**Grapefruit**	*Citrus* x *paradisi*	Rio Red	Permissive	Host
**Sweet orange**	*Citrus* x *sinensis*	Marrs	Permissive	Host
**Lemon**	*Citrus* x *limon*	Valley lemon	Permissive	Host
**Curry leaf**	*Murraya koenigii*	Unknown	Non-permissive	Host
**Orange jasmine**	*Murraya exotica*	Lakeview	Non-permissive	Host
**White sapote**	*Casimiroa edulis*	Unknown	Non-permissive	Non-host
**Madagascar periwinkle**	*Catharanthus roseus*	Unknown	Permissive	Non-host

^a^Permissive, supports CLas growth and multiplication; Non-permissive, suppress *C*Las growth and multiplication.

^b^Host, feeding and reproductive host of the ACP; Non-host, not colonized by the ACP.

### Phloem sap collection

Phloem saps were extracted from young expanding flush shoots and mature shoots of *Citrus* and non-*Citrus* rutaceous plants using the ethylenediaminetetraacetic acid (EDTA) method according to [26] with some modification [20]. Briefly, flush shoots were excised at the point of attachment to the main twig using sterilized pruning shears and immediately immersed into 30 mL of 20 mM EDTA solution in plastic vials. Whole periwinkle plants cut at their base were processed in a similar manner. The vials were then covered with moist paper towels and transported on dry ice to the laboratory to maintain sample integrity. Samples were agitated at 100 rpm on a table shaker in a dark, temperature (21°C) controlled room for 3 hrs as described by Sétamou et al. [20] Ten young flush shoots, four mature shoots of Rutaceae and two whole periwinkle plants were used for phloem sap collection and three replicates of each sample were prepared. Excised flush shoots or periwinkle plants were removed from the tubes and the EDTA solution with phloem sap extracts was transferred into sterile 50 mL centrifuge tubes and stored at -80°C until further processing. The mass of each cut rutaceous flush shoot or periwinkle plant was individually measured using a Mettler Toledo MS104S analytical balance (Mettler Toledo Inc., Greifensee, Switzerland). Prior to free amino acid analysis, tubes containing the frozen phloem sap solutions were retrieved, uncapped, and freeze-dried for 96 hrs. using a benchtop lyophilizer. The freeze-dried EDTA-phloem exudate (Millrock Bench-Top Freeze-Dryer BT48, Millrock Technology, Kingston, NY) were analyzed for amino acid contents at the University of Missouri-Columbia Experimental Station Chemical Laboratories, following previously described procedures [20].

### Extraction of *D*. *citri* amino acid

Forty live *D*. *citri* nymphs and an equal number of adults were collected from a laboratory-reared colony and thoroughly homogenized separately in 15 ml of EDTA in centrifuge tubes. The homogenates were filtered using a Whatman #2 filter paper, and aliquots (~12 ml) of supernatants were centrifuged at 14,000 × g for 5 min. Approximately 10 ml of the supernatant was collected, freeze-dried, and analyzed for amino acid composition as described above. The analyses involved three replicates of each *D*. *citri* developmental stage. In addition, the mass of a group of 10 adults or nymphs (n = 10) was measured using the analytical scale and the mass values were used to determine free amino acid contents of *D*. *citri* nymphs and adults per gram of body mass.

### Statistical analyses

The FAA concentrations in the various plant phloem exudates and whole psyllid samples were calculated by normalizing the level of each FAA per gram of fresh tissue or whole insect per replicate. Mean values were determined for each FAA per host. A Venn diagram was used to compare the FAA composition of the different hosts based on their presence or absence in the sample (http://bioinformatics.psb.ugent.be/webtools/Venn/). An agglomerative hierarchical cluster (ACH) analysis computed with the Euclidian distance as proximity type and the Ward’s agglomeration method with automatic entropy truncation [27] was used to classify the different hosts in a limited number of relatively homogenous groups according to their amino acid profiles. Grouping of FAAs was similarly performed via ACH, and a heatmap was used to graphically represent the relationship between FAA profiles and host clusters generated based on relative FAA concentrations. In addition, a partial least square discriminant analysis (PLS-DA) that generates a supervised pattern recognition matrix was used to extract maximum information on discriminant compounds for the data. Using PLS-DA, the most discriminatory FAAs between *C*Las -permissive hosts and non-permissive plants were identified using the index scores of their variable importance on the projection (VIP) plot. An FAA with a VIP > 1 score is considered important [28]. The Pearson linear correlation was used to determine relationships between individual FAAs and to identify those FAAs which highly correlated with the host *C*Las host status. The relative ratios of each FAA between *C*Las -permissive and non-permissive hosts as determined by the PLS-DA were generated, and these ratios were compared to 1 using the Student’s *t*-test. All analyses were performed with the XLSTAT software (version 2016, Addinsoft, Paris, France).

## Results

Mean values of total amino acids detected in the phloem sap of young and mature shoots of the six Rutaceae plants, whole periwinkle and whole *D*. *citri* adults and nymphs (Table 1) are presented in Table 2. More individual FAAs were detected in citrus phloem (35) relative to *D*. *citri* nymphs (30) and adults (31), periwinkle (25) and non-citrus Rutaceae (19–28). Across plant species, between one and nine additional FAAs were detected in the phloem sap of young shoots compared to mature shoots. Similarly, significantly higher concentrations of total and individual FAAs were detected in phloem sap of young shoots of each host plant compared to mature shoots of the same plants (5-39-fold range; Table 2) and this disparity was greater in *C*Las -permissive hosts (21-39-fold range) than in non-permissive hosts (5-10-fold range). The total amount of amino acids detected per gram of tissue in *D*. *citri* 5^th^ instars was comparable to that detected in equivalent numbers of adults.

**Table 2:** Mean amino acid concentration^1^(μg/g) of leaf tissue of *Candidatus* Liberibacter asiaticus (*C*Las)-permissive and non–permissive plants^2^ and of whole *Diaphorina citri* (ACP) adults and nymphs analyzed by HPLC.

		*C*Las-permissive hosts									*C*Las-non-permissive hosts					
		Grapefruit		Lemon		Sweet orange		ACP^3^		Peri-winkle^4^	Curry leaf		Orange jasmine		White sapote	
	Code	Y	M	Y	M	Y	M	Ny	Ad	Stem	Y	M	Y	M	Y	M
Mass per sample unit in grams ± SE		1.4 ±0.1	3.1 ±0.1	0.5 ±0.1	2.4± 0.0	0.6 ± 0.0	3.5± 0.0	0.00013 ±0.00002	0.00055 ±0.00009	2.9 ± 0.5	0.1 ±0.0	0.8 ±0.0	0.3 ±0.0	1.6 ±0.1	1.3 ±0.0	3.5 ±0.3
**Free amino acid**																
Phosphoserine	SEP	1.4	0.4	2.2	0.8	2.5	0.5	475.0	237.5	0.9	6.1	1.8	4.1	1.5	0.7	0.6
Taurine	TAU	0.9	-	1.1	-	1.1	-	-	37.5	-	-	-	-	-	-	-
Phosphoethanolamine	PHOS	1.8	0.8	3.1	1.2	3.4	0.8	150.0	56.3	0.3	13.9	4.7	10.9	3.9	1.3	1.2
Aspartic Acid	ASP	0.6	0.2	1.9	0.4	1.8	0.1	800.0	275.0	12.6	2.0	0.2	1.0	-	0.6	-
Hydroxyproline	HYP	0.4	-	2.1	-	1.2	-	-	-	0.2	-	-	-	-	-	-
Threonine	THR	4.3	0.1	7.9	0.3	7.8	0.1	1175.0	1075.0	0.9	15.7	1.6	10.1	0.3	1.7	0.2
Serine	SER	14.7	0.4	28.8	1.2	26.8	0.7	1050.0	343.8	3.8	11.8	1.1	6.2	0.3	1.2	0.2
Asparagine	ASN	30.6	3.3	116.1	3.1	45.4	0.7	1625.0	425.0	2.3	12.4	0.6	9.6	0.6	1.3	-
Glutamic Acid	GLU	1.3	0.2	5.3	0.8	4.1	0.3	2125.0	1162.5	3.8	6.5	0.5	6.3	0.3	2.5	0.4
Glutamine	GLN	3.1	0.3	4.8	0.6	10.8	0.3	2825.0	3143.8	1.1	-	-	1.9	-	0.9	-
Sarcosine	SAR	2.0	-	2.0	0.1	2.1	0.1	100.0	18.8	-	-	-	0.6	-	0.8	-
α-amino-adipic acid	AAD	2.8	-	1.9	0.1	1.7	-	75.0	25.0	-	0.2	-	1.0	-	0.1	-
Proline	PRO	73.4	3.2	168.1	6.3	141.8	4.7	3600.0	3037.5	11.4	2.5	0.1	13.7	0.2	0.2	-
Glycine	GLY	2.9	0.1	5.9	0.2	4.4	0.1	575.0	168.8	1.7	9.7	1.3	2.6	0.5	0.4	0.2
Alanine	ALA	19.3	0.2	31.9	0.6	28.7	0.3	2025.0	725.0	1.3	8.4	0.8	3.4	0.2	1.8	0.1
Citrulline	CIT	-	-	-	-	0.4	-	-	-	-	1.2	0.1	-	-	-	-
α-amino-n-butyric acid	AABA	0.4	-	0.4	-	0.5	-	25.0	31.3	0.1	0.6	0.2	0.5	0.1	0.1	-
Valine	VAL	3.6	0.1	5.9	0.3	8.5	0.2	650.0	206.3	0.7	2.6	0.3	0.8	0.1	0.2	-
Methionine	MET	0.7	-	1.0	-	1.7	-	75.0	106.3	0.1	-	-	-	-	-	-
Cystine	CYS	0.2	-	0.1	-	0.3	-	100.0	18.8	1.2	-	-	-	-	-	-
Isoleucine	L-CYS2	1.9	-	2.7	0.1	4.1	0.1	275.0	56.3	-	1.4	-	0.2	-	0.1	-
Leucine	LEU	4.0	0.1	5.0	0.2	7.2	0.1	250.0	81.3	-	1.6	0.1	0.5	-	0.1	-
Tyrosine	TYR	4.1	0.1	6.3	0.1	5.9	0.1	1575.0	268.8	-	3.3	0.5	1.4	0.3	0.3	0.1
Cystathionine	CYSTA	-	-	-	-	-	-	25.0	50.0	-	-	-	-	-	-	-
Phenylalanine	PHE	2.1	0.1	3.6	0.1	5.3	0.1	300.0	68.8	0.5	1.5	0.2	0.6	-	0.1	-
β-alanine	β-ALA	1.5	-	1.4	-	2.6	-	75.0	18.8	-	-	-	1.4	-	0.2	-
β-amino-isobutyric acid	BAIBA	0.1	-	4.4	-	-	-	-	18.8	-	-	-	-	-	-	-
γ-amino-butyric acid	GABA	27.9	0.4	48.6	1.0	51.9	0.6	100.0	37.5	9.5	1.2	0.1	4.1	0.3	0.8	0.1
Homocysteine	HCY	-	-	-	-	-	-	75.0	50.0	-	-	-	-	-	-	-
Ethanolamine	ETA	1.9	0.1	2.5	0.2	3.4	-	-	-	-	-	-	-	-	-	-
Tryptophan	TRP	3.4	-	6.3	-	1.8	-	950.0	-	-	-	-	-	-	-	-
Hydroxylysine	HYL	0.1	-	0.1	-	0.3	-	-	-	-	-	-	-	-	-	-
Ornithine	ORN	0.2	-	0.4	-	0.5	-	250.0	81.3	0.3	7.8	0.8	1.5	0.1	0.1	0.1
Lysine	LYS	3.5	0.1	4.7	0.2	8.6	0.2	500.0	131.3	0.4	2.3	0.2	0.9	0.1	0.3	-
1-methyl-histidine	1-MHIS	1.3	-	1.3	-	2.6	-	-	-	-	-	-	-	-	-	-
Histidine	HIS	1.5	0.1	2.6	0.1	3.5	0.1	950.0	418.8	0.4	4.2	0.4	1.0	0.1	1.5	0.1
3-methyl-histidine	3-MHIS	0.1	-	0.1	-	0.1	-	-	-	-	-	-	-	-	-	-
Carnosine	CAR	-	-	-	0.4	-	0.1	-	-	-	-	-	-	-	-	-
Arginine	ARG	4.8	0.6	8.2	0.9	1-	0.2	1675.0	800.0	0.4	3.7	0.4	0.9	0.1	0.2	-
Total FAA		223	11	489	19	403	10	3843	3711	54	121	16	85	9	17	3
Proteinogenic AA (PAA)		180	9	417	15	328	8	3631	3518	43	90	9	61	3	13	1
^6^Ratio PAA: FAA (%)		80.8	83.9	85.4	80.4	81.5	79.5	94.5	94.8	79.3	74.3	52.3	71.6	33.9	77.3	39.5
% Change with age		3.1		-5.0		-2.0		0.3		NC^5^	-22.0		-37.7		-37.8	

^1^ Mean concentration based on 3 replications per sample.

^2^ For Rutaceae host plants, phloem sap of young (Y) and mature (M) flush shoots were tested.

^3^ For *D*. *citri* a sample whole samples of 40 nymphs (Ny) and 40 adults (Ad) were pooled and tested as a replicate.

^4^ Phloem sap was collected from two whole periwinkle plants cut at the base per sample.

^5^ NC = not calculated.

^6^ Ratio of Proteinogenic AA to total FAA.

Several proteinogenic and non-proteinogenic FAAs were detected in whole *D*. *citri* nymphs and adults and in phloem sap extracts of *C*Las permissive and non-permissive plants (Table 2). All *C*Las -permissive plant species (Table 1) and *D*. *citri* life stages (nymph and adult) had greater proportions of proteinogenic FAAs (≈80–95%) compared to *C*Las -tolerant/resistant plants (≈34–77%) (Table 2). The total AA pool of the three *Citrus* species was dominated (79.5–85.4%) by proteinogenic FAAs regardless of flush shoot maturity status (young vs. old) (Table 2). Adult and immature *D*. *citri* samples contained comparably higher numbers of proteinogenic FAAs that represented most (≈95%) of their entire AA pools. Similarly, the AA pool of the *C*Las-permissive periwinkle was abundant in proteinogenic FAA (79.3%). Although 72–77% of total FAAs in young shoots of non-*Citrus* rutaceous plants (curry leaf, orange jasmine and white sapote) were proteinogenic, non-proteinogenic FAAs were the most abundant in phloem sap of mature shoots of these plants with the exception of curry leaf (Table 2). While the proportion of proteinogenic FAAs did not vary with flush shoot growth stage in *Citrus* spp. and between *D*. *citri* developmental stages, there was a dramatic decrease in the relative concentrations of this class of FAAs as flush shoots matured in *C*Las-tolerant/resistant non-*Citrus* rutaceous plants (Table 2).

**Fig 1:**
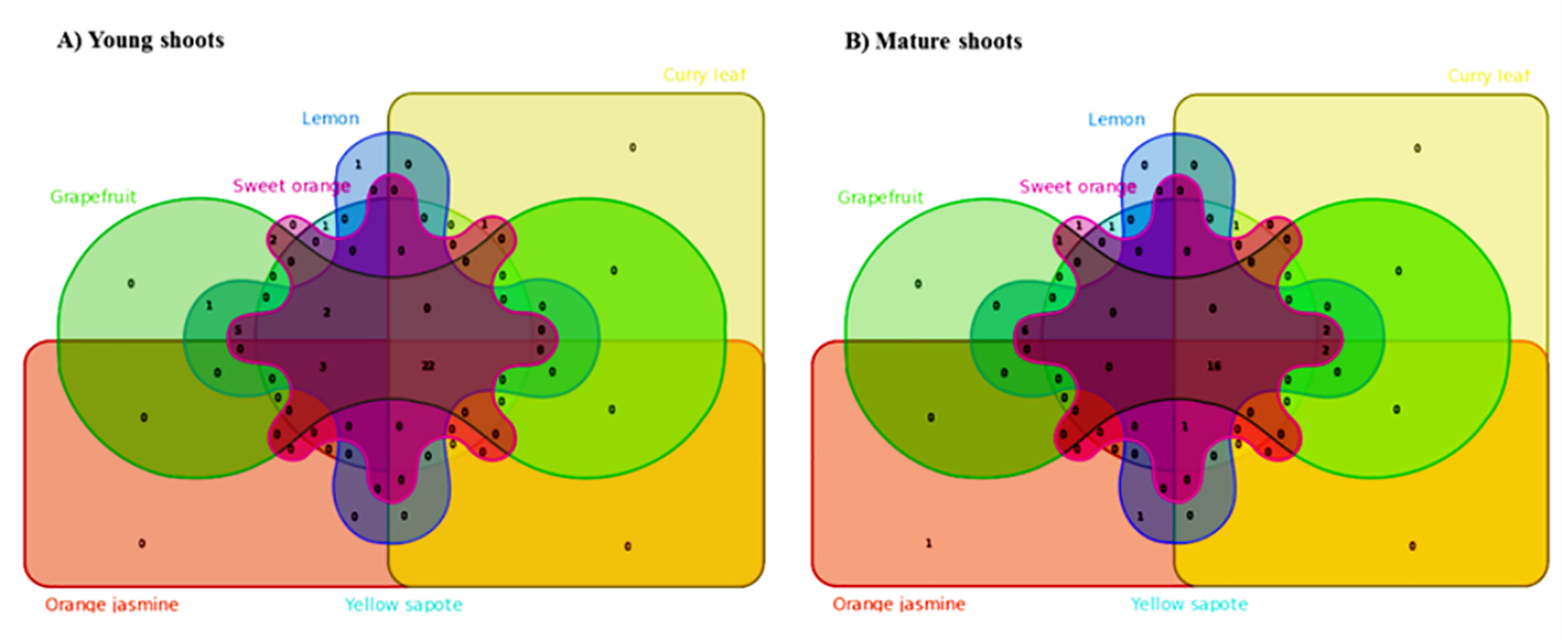
Venn diagram comparing shared and unique free amino acids present in phloem sap of non-permissive (curry leaf, orange jasmine and white sapote), and permissive (grapefruit, lemon and sweet orange) hosts of *Candidatus* Liberibacter asiaticus (*C*Las). A = young shoots and B = mature shoots.

A Venn diagram was used to compare the FAA profiles between the different hosts (Fig 1). Eighteen (18) FAAs (13 proteinogenic: alanine, arginine, asparagine, aspartate, glutamate, glycine, histidine, lysine, phenylalanine, proline, serine, threonine and valine; and five non-proteinogenic: α- amino butyric acid [AAAB], γ-amino butyric acid [GABA], ornithine, phosphoethanolamine and phosphoserine) were common to *D*. *citri* (adults and nymphs), periwinkle, and young shoots of all *Rutaceae* (S1 Table). Phloem sap of young shoots of citrus and periwinkle plants contained cystine that was not detected in sap of young shoots of curry leaf, orange jasmine and white sapote. In addition, tryptophan, hydroxylysine, taurine, carnosine and 1-methyl-histidine were present in phloem sap of young citrus shoots, but absent from sap of young shoots of non-citrus Rutaceae (S1 Table). No FAA was found exclusively in young shoot phloem sap of *C*Las-tolerant/resistant Rutaceae. With mature shoots, there were 15 FAAs shared by all experimental hosts (Fig 1, S2 Table). Methionine and glutamine were present in phloem sap of mature shoots of citrus, periwinkle, and in whole *D*. *citri* (nymphs and adults) but absent in phloem sap of mature shoots of *C*Las-tolerant/resistant plants. A comparative analysis of mature shoots of rutaceous plants showed that phloem sap of *C*Las-permissive citrus hosts contained five FAA (glutamine, methionine, carnosine, α-amino-adipic acid and ethanolamine) that were not detected in non-citrus rutaceous plants (Fig 1). With the exception of orange jasmine, citrulline was present only in mature shoots of *CLas* non-permissive plants (Fig 1).

**Fig 2:**
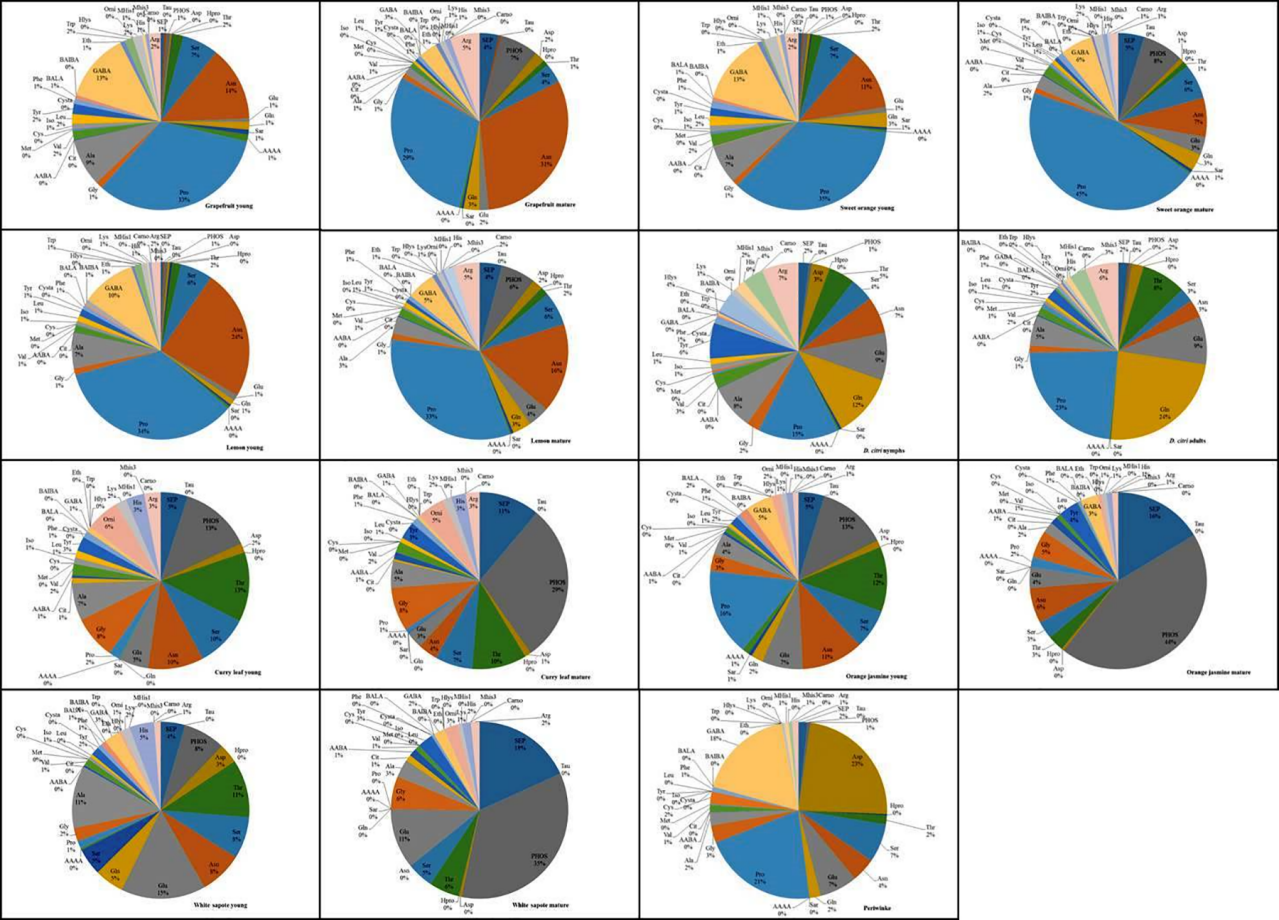
Relative concentrations of free amino acids detected in phloem sap of young and mature Rutaceae flush shoots, periwinkle plants, and whole *Diaphorina citri* nymphs and adults populations.

Concentrations of each AA expressed in micrograms per gram of host tissue (μg/g) were subjected to multivariate analysis. Although *C*Las-permissive hosts and non-permissive plants shared several FAAs in common, the relative concentrations of individual FAAs varied with the host type, and growth/developmental stage. In general, proline, GABA, aspartate, glutamate, asparagine and glutamine were the most abundant FAAs in all *C*Las-permissive plants (Fig 2). The six FAAs collectively represented 61 to 71% of the total FAA pool of citrus flush shoot phloem sap and showed no significant variation as the shoots matured. These six FAAs were also dominant in *D*. *citri* nymphs (45%), adults (61%) and periwinkle (75%). In contrast, all six FAAs constituted only 9–42% of phloem sap of *C*Las non-permissive Rutaceae plants (Fig 2). Interestingly, a 2 to 3-fold decrease in total concentrations of the six most abundant FAAs present in permissive hosts was observed in phloem sap of *C*Las-tolerant/resistant plants with flush shoot maturity (young shoot = 20–42% vs. mature shoot = 9–16%) whereas no such changes occurred between young and mature shoots of *C*Las-permissive citrus plants. Notably, though abundant in *D*. *citri*, glutamine was absent in phloem sap of mature shoots from *C*Las non-permissive plants and in young shoots of curry leaf. The phloem sap AA pool of CLas tolerant/resistant plants was dominated by glycine, serine, threonine phosphoserine, phosphoethanolamine and ornithine with their total concentrations ranging from 31% in young flush shoots of white sapote, to 73% in mature shoots of orange jasmine (Fig 2). In contrast, these six FAAs abundant in *C*Las tolerant/resistant plants constituted only 10–21% of total FAA content of *C*Las-permissive hosts (Fig 2).

**Fig 3:**
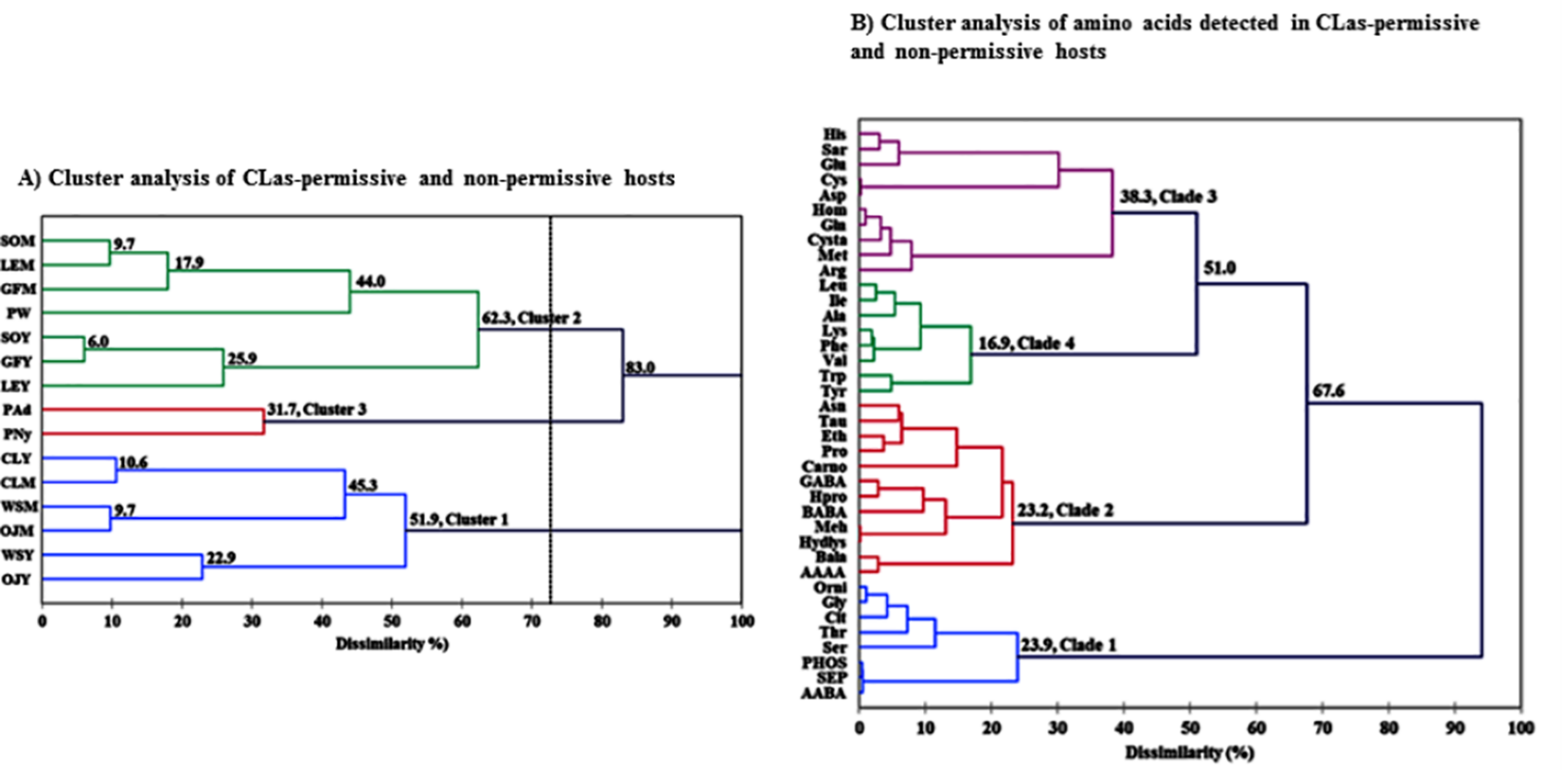
**Agglomerative hierarchical cluster (ACH); A. Dendrogram depicting clustering patterns of *C*Las-permissive and non-permissive hosts of *Candidatus* Liberibacter asiaticus (*C*Las) into similar groups based on their relative amino acid profiles.** Young (Y) and mature (M) flush shoots of were tested for each Rutaceae plant species. SO, sweet orange; GF, grapefruit; LE, lemon; PW, periwinkle plant; CL, curry leaf; OJ, orange jasmine; WS, white sapote; PAd, psyllid adults; PNy, psyllid nymphs. B. Dendrogram depicting clades formed by various free amino acids based on their relative concentrations in the various hosts tested.

The agglomerative hierarchical cluster (ACH) analysis was used to group the different hosts into three clusters based on their FAA profiles (Fig 3). Cluster 1 comprised both flush growth stages (young and mature) of all three non-citrus plants (curry leaf, orange jasmine and white sapote), and had ≈52% dissimilarity in their amino composition. All *C*Las-permissive plants (*Citrus* spp. and periwinkle) segregated into cluster 2 (Fig 3). Within this cluster, phloem sap FAA profiles of mature shoots of *Citrus* spp. were more similar to that of periwinkle while young shoot phloem sap FAA profiles of the three *Citrus* species were more similar (only ≈26% dissimilarity). The two life stages of *D*. *citri* (nymphs and adults) segregated into cluster 3 with ≈32% dissimilarity between their FAA profiles. Hence, clusters 1, 2 and 3 were designated as non-citrus, citrus and psyllid clusters, respectively. FAA-based ACH analysis resulted in the identification of four distinct clades. Clade 1 contained FAAs that were dominant in *C*Las-tolerant/resistant plants (e.g. AAAB, phosphoserine, phosphoethanolamine, serine, threonine, citrulline, glycine and ornithine), while Clades 2 and 3 comprised most FAAs present in relatively higher concentrations in *C*Las-permissive hosts (e.g. arginine, proline, GABA, aspartate, asparagine, glutamate and glutamine and the sulfur-containing FAA methionine, cystine and taurine) (Fig 3). Clade 3 specifically grouped together FAAs that were the most abundant in *D*. *citri* and periwinkle plants such as arginine, aspartate, glutamate, glutamine, methionine and cysteine, among others (Fig 3). Clade 4 included FAAs that were present in relatively higher concentrations in *D*. *citri* nymphs and moderately present in young flush shoots of Rutaceae plants relative to mature shoots, including alanine, lysine, leucine, isoleucine, phenylalanine, valine, tryptophan and tyrosine.

**Fig 4:**
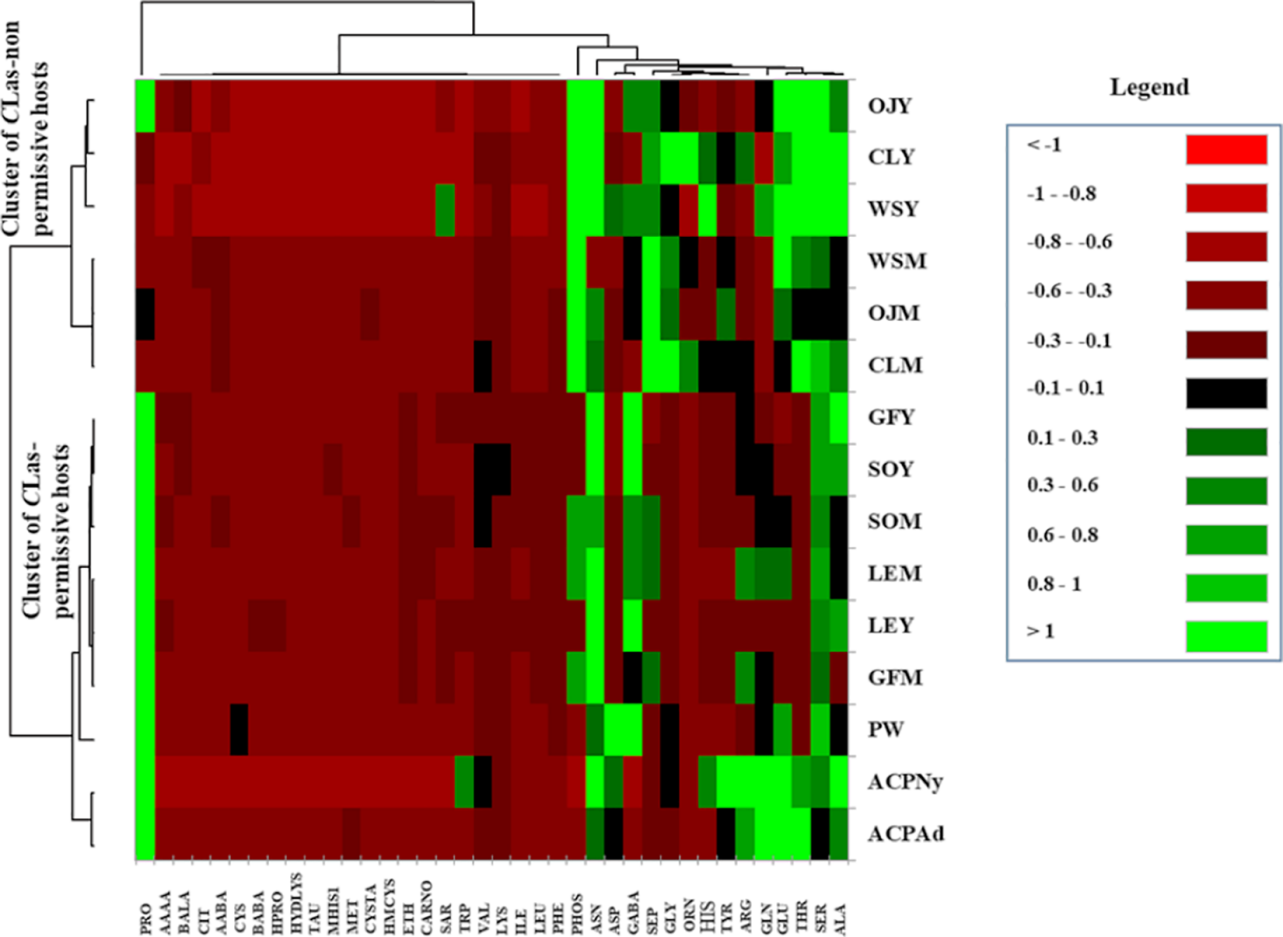
Cluster heatmap describing the relative concentrations of free amino acids in phloem sap of permissive hosts of *Candidatus* Liberibacter asiaticus, non-permissive plants and whole *Diaphorina citri* nymphs and adults (OJY = young orange jasmine, OJM = mature orange jasmine, CLY = young curry leaf, CLM = mature curry leaf, WSY = young white sapote, WSM = mature white sapote, GFY = young grapefruit, GFM = mature grapefruit, SOY = young sweet orange, SOM = mature sweet orange, LEY = young lemon, LEM = mature lemon, PW = periwinkle plant, ACPAd = *D*. *citri* adults, and ACPNy = *D*. *citri* nymphs).

**Fig 5:**
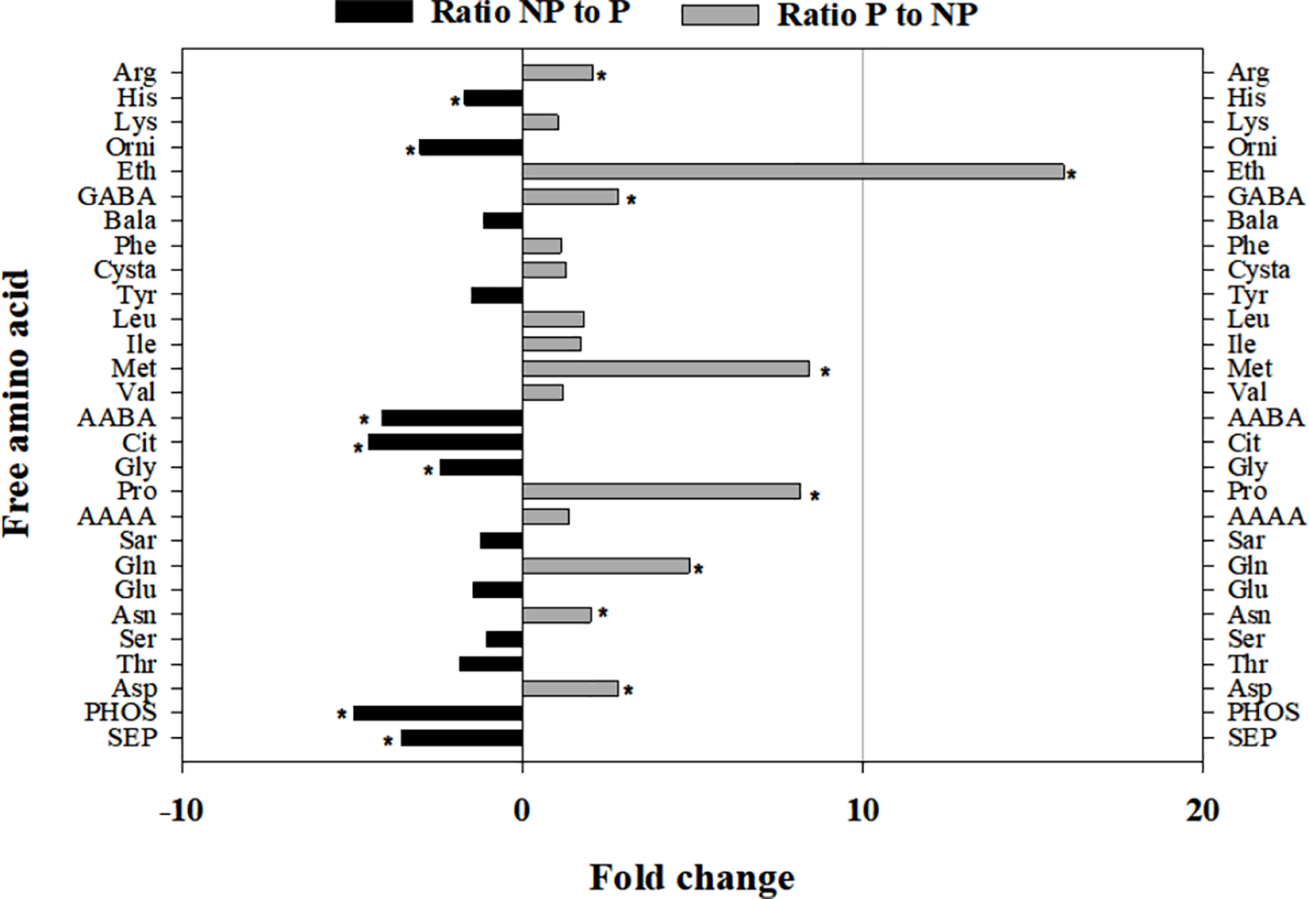
Free amino acid profiles of permissive hosts (P) of *Candidatus* Liberibacter asiaticus (*C*Las) and non-permissive (NP) plants. Permissive hosts comprise phloem sap of young and mature flush shoots of three citrus species, periwinkle plants and whole *Diaphorina citri* nymphs and adults, while non-permissive host comprise young and mature shoots of curry leaf, orange jasmine and white sapote. Results are based on mean values obtained for growth stages of each plant species (three replicates) and are shown as the ratios of accumulation between the two host categories. Asterisk indicates significant differences according to t-test (P<0.05).

A heat map describing the association between FAA content of the different hosts was generated. To enhance readability of the heat map, FAAs with low variability (i.e. interquartile range less than 0.05) were removed from the analysis. The heat map grouped the different hosts into two clusters that strongly correlated with their CLas permissivities (Fig 4). With the exception of young shoot phloem sap of orange jasmine, all CLas-permissive hosts had higher proline concentrations relative to non-permissive plants. Similarly, *C*Las permissive hosts tended to have higher concentrations of arginine, glutamine and GABA. In contrast, *C*Las non-permissive plants had higher concentrations of phosphoserine, phosphoethanolamine, glycine, and ornithine relative to CLas-permissive hosts (Fig 4). The ratios of individual AAs were calculated to evaluate the variation in FAA between the two clusters of *C*Las permissive and non-permissive hosts (Fig 5). Notably, α-amino-n-butyric acid, citrulline, glycine, ornithine, phosphoserine and phosphoethanolamine were present in significantly higher concentrations in *C*Las non-permissive hosts relative to permissive ones. In contrast, *C*Las-permissive hosts contained 3 to 16-fold more arginine, asparagine, aspartic acid, ethanolamine, GABA, glutamine, hydroxyproline, proline and the sulfur containing amino acid methionine.

**Fig 6:**
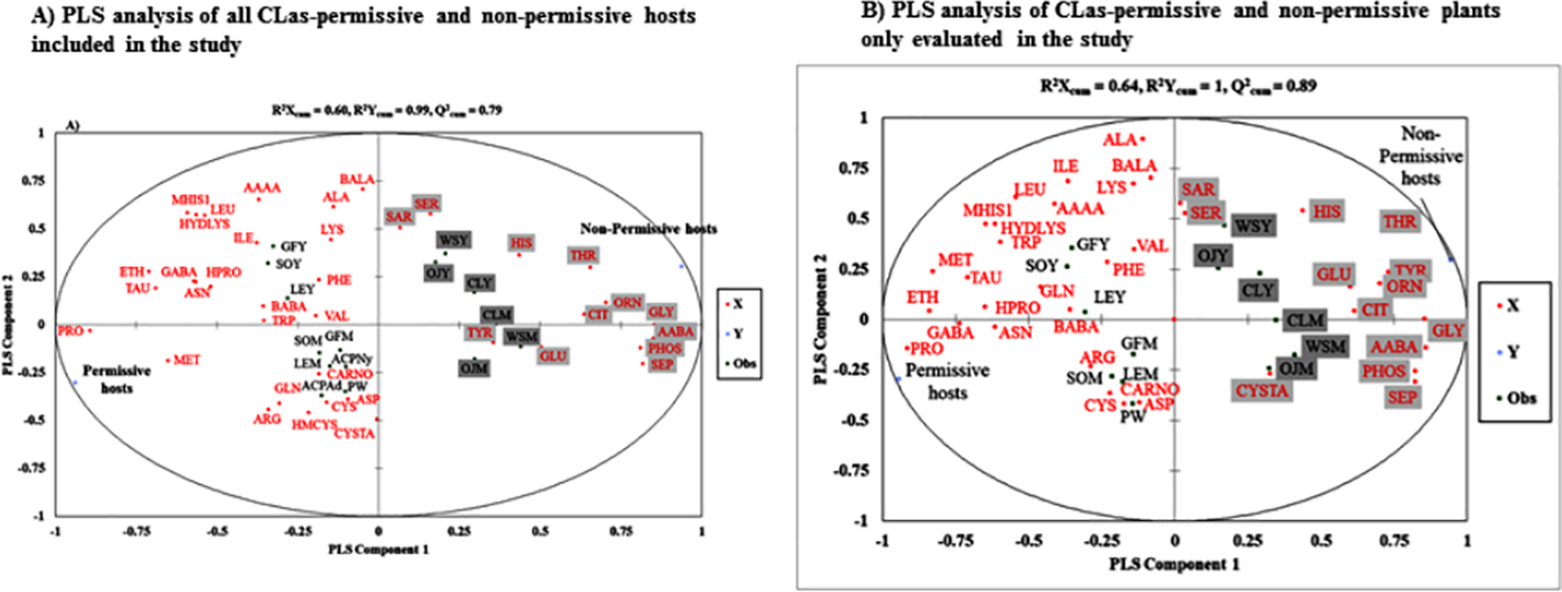
Partial least squares-discriminant analysis (PLS-DA). Correlations between permissive and non-permissive hosts of *Candidatus* Liberibacter asiaticus (*C*Las) and their free amino acid content as explanatory variables (X). The ellipse represents the Hotelling T^2^ with 95% confidence interval (R^2^X_cum_ = 0.58, R^2^Y_cum_ = 0.99, Q^2^_cum_ = 0.79 for all hosts tested (A); R^2^X_cum_ = 0.64, R^2^Y_cum_ = 1, Q^2^_cum_ = 0.89 for *C*Las-permissive and non-permissive plants only (B). Q^2^_cum_ = cumulative fraction of the total variation of X’s that can be predicted by the extracted components; R^2^X_cum_ and R^2^Y_cum_ represent the fraction of the sum of squares of all X’s and Y’s explained by the current components, respectively. P = *C*Las-permissive and NP = *C*Las-non-permissive hosts defined as: OJY = young orange jasmine, OJM = mature orange jasmine, CLY = young curry leaf, CLM = mature curry leaf, WSY = young white sapote, WSM = mature white sapote, GFY = young grapefruit, GFM = mature grapefruit, SOY = young sweet orange, SOM = mature sweet orange, LEY = young lemon, LEM = mature lemon, PW = periwinkle plant, ACPAd = *D*. *citri* adults, and ACPNy = *D*. *citri* nymphs. The amino acids are defined as: AAAA, α-amino adipic acid; AABA, α-aminobutyric acid; Ala, alanine; Arg, arginine; Asn, asparagine; Asp, aspartate; BABA, β-aminobutyric acid; BALA, β-alanine; Carno, carnosine, Cit, citrulline; Cys, cystine; Cysta, cystathionine; Eth, ethanolamine; GABA, γ-aminobutyric acid; Gln, glutamine; Glu, glutamate; Gly, glycine; His, histidine; Hom, homocysteine; Hpro, hydroxyproline; Hydlys, hydroxyl-lysine; Ile, isoleucine; Leu, leucine; Lys, lysine; Meh, 1-methyl histidine; Met, methionine; Orn, ornithine; Phe, phenylalanine; PHOS, phosphoethanolamine; Orn, ornithine, Pro, proline; Sar, sarcosine; SEP, phosphoserine; Ser, serine; Tau, taurine; Thr, threonine; Trp, Tryptophan; Tyr, tyrosine; Val, valine.

A supervised pattern recognition method or partial least squares-discriminant analysis (PLS-DA) was also used to visualize differences in FAA profiles between the various hosts, between *C*Las-permissive hosts and non-permissive plants, and to identify specific FAAs that are related to *C*Las permissivity. A clear discrimination was obtained between *C*Las permissive and non-permissive hosts with the PLS-DA analysis along the first PLS component (Fig 6), indicating effective removal of FAA variation not correlated to the two CLas response categories. The model discriminating the two *C*Las response categories had good predictability as shown by a high quality index (cumulative Q^2^ = 0.774). The cumulative R^2^Y and R^2^X that correspond to the correlation between the FAAs (parameter X) and CLas permissivity group (parameter Y) with the PLS components were 0.58 and 0.99, respectively. Simialr analysis using phloem sap FAA of tested plants resulted in similar results (Fig 6). Interestingly, *C*Las -permissive and non-permissive plants separated quite along PLS component 1, while PLS component 2 discriminated young and mature flush shoots of both types of hosts (Fig 6). Hence both the FAA and host data were well summarized by the four components generated with the PLS-DA analysis (S1 Fig). The FAAs with high variable importance in projection scores were regarded as contributing most significantly to *C*Las host discrimination. In this regard, twelve (12) FAAs had VIP scores of >1 indicating their importance in discriminating *C*Las -host status (S1 Fig). Based on their correlation values with *C*Las -host response categories (Table 3, S1 Fig), these 12 FAAs can be classified as positively (arginine, ethanolamine, methionine, proline, and taurine) or negatively (α-amino-n-butyric acid, β-alanine, citrulline, glycine, ornithine, phosphosethanolamine, phosphoserine and threonine) associated with *C*Las growth and multiplication. The proportion of all FAAs positively correlated *C*Las susceptibility in *C*Las-permissive hosts was 41.9% compared to only 8.1% in *C*Las-non-permissive plants, suggesting that these FAAs may be positively related to *C*Las growth. In contrast, non-permissive plants had 5.5-fold more FAAs negatively correlated with *C*Las growth relative to permissive hosts (52% vs 9.5%). Using the standardized coefficients of the model relating FAAs to *C*Las host susceptibility (S1 Fig), it was observed that only four FAA namely, GABA, ethanolamine, methionine and proline were positively and significantly related to *C*Las-susceptibility with 95% confidence limits not encompassing 0. Similarly, six FAAs, AABA, glycine, ornithine, phosphoethanolamine, phosphoserine and threonine were the only amino acids significantly related to *C*Las non-permissivity. *C*Las-permissive hosts were characterized by significantly higher proline to glycine, proline to AABA and proline to threonine ratios as compared to non-permissive hosts (Table 4). However, using the 95% jackknife confidence interval as implemented in the PLS-VIP method (Blasco et al. 2015), only two FAAs namely proline and glycine had lower boundaries that did not encompass 1, suggesting that these two variables may be the primary discriminating factors between *C*Las-permissive and non-permissive hosts. Since these two FAAs were negatively correlated (*r* = -0.82, *P*< 0.001, Table 3), the derived proline to glycine ratios varied significantly with *C*Las-host status with lower ratios in *C*Las-non-permissive relative to *C*Las-permissive hosts (Table 4).

**Table 3:** Correlation between mean amino acid concentrations and *Candidatus* Liberibacter asiaticus (*C*Las) permissivity status of different plants and *Diaphorina citri* (ACP) adults and nymphs.

Variables	SEP	TAU	PHOS	ASP	HPRO	THR	SER	ASN	GLU	GLN	SAR	AAAA	PRO	GLY	ALA	CIT	AABA	VAL	MET	CYS	ILE	LEU	TYR	CYSTA	PHE	BALA	BABA	GABA	HMCYS	ETH	TRP	HYDLYS	ORN	LYS	MHIS1	HIS	CARNO	ARG	NP	P
SEP	**1.00**		** **																																				** **	** **
TAU	-0.48	**1.00**																																					** **	** **
PHOS	**0.97**	-0.45	**1.00**																																				** **	** **
ASP	-0.23	-0.22	-0.26	**1.00**																																			** **	** **
HPRO	-0.47	0.38	-0.44	0.32	**1.00**																																		** **	** **
THR	0.19	-0.37	0.22	-0.16	-0.47	**1.00**																																	** **	** **
SER	-0.23	-0.32	-0.20	0.17	0.19	0.38	**1.00**																																** **	** **
ASN	-0.44	**0.56**	-0.37	-0.21	0.29	-0.39	-0.05	**1.00**																															** **	** **
GLU	0.23	-0.49	0.11	0.21	-0.43	**0.53**	-0.04	**-0.57**	**1.00**																														** **	** **
GLN	-0.34	0.26	-0.40	0.01	-0.21	0.14	**-0.52**	-0.21	0.37	**1.00**																													** **	** **
SAR	-0.19	-0.07	-0.19	-0.07	-0.11	0.23	0.21	0.01	0.50	0.03	**1.00**																												** **	** **
AAAA	-0.45	0.41	-0.38	-0.27	0.12	0.04	0.22	0.35	-0.23	-0.06	0.20	**1.00**																											** **	** **
PRO	**-0.66**	**0.53**	**-0.66**	-0.01	0.45	**-0.69**	-0.08	0.50	-0.59	0.14	-0.16	0.36	**1.00**																										** **	** **
GLY	**0.67**	**-0.53**	**0.69**	-0.01	-0.33	**0.61**	0.32	-0.47	0.19	-0.37	-0.24	-0.41	**-0.82**	**1.00**																									** **	** **
ALA	-0.42	0.16	-0.42	-0.21	0.17	0.35	0.32	-0.09	0.26	0.21	**0.59**	0.32	-0.13	-0.08	**1.00**																								** **	** **
CIT	0.55	-0.29	0.46	-0.16	-0.23	0.44	0.31	-0.34	0.23	-0.30	-0.25	-0.34	**-0.55**	0.75	-0.01	**1.00**																							** **	** **
AABA	**0.96**	-0.45	**0.96**	-0.25	-0.41	0.36	-0.12	-0.51	0.20	-0.33	-0.20	-0.36	**-0.73**	**0.78**	-0.29	**0.58**	**1.00**																						** **	** **
VAL	-0.36	-0.08	-0.36	-0.10	-0.01	0.16	0.30	-0.26	-0.11	0.25	-0.12	-0.05	0.10	0.10	0.49	0.08	-0.25	**1.00**																					** **	** **
MET	**-0.57**	**0.55**	**-0.62**	0.05	0.20	-0.19	-0.38	-0.08	0.05	**0.84**	0.07	0.08	0.49	**-0.61**	0.31	-0.42	**-0.56**	0.29	**1.00**																				** **	** **
CYS	-0.24	-0.17	-0.27	**0.98**	0.39	-0.23	0.12	-0.24	0.14	0.03	-0.17	-0.24	0.04	-0.03	-0.20	-0.17	-0.25	-0.03	0.10	**1.00**																			** **	** **
ILE	**-0.56**	0.18	**-0.55**	-0.26	0.18	0.10	0.36	0.05	-0.09	0.19	0.14	0.31	0.22	-0.18	**0.74**	0.03	-0.50	**0.74**	0.34	-0.20	**1.00**																		** **	** **
LEU	**-0.59**	0.50	**-0.53**	-0.39	0.33	-0.04	0.37	0.30	-0.51	0.01	0.00	0.51	0.43	-0.24	**0.60**	-0.06	-0.47	**0.64**	0.30	-0.32	**0.81**	**1.00**																	** **	** **
TYR	0.33	-0.27	0.34	-0.30	-0.35	0.30	-0.26	-0.39	0.24	0.20	-0.14	-0.15	**-0.52**	0.41	0.32	0.23	0.38	0.47	-0.08	-0.22	0.31	0.06	**1.00**																** **	** **
CYSTA	0.14	0.13	0.16	-0.09	-0.26	0.08	**-0.69**	-0.32	0.19	**0.73**	-0.20	-0.24	-0.12	-0.06	-0.09	-0.22	0.13	-0.02	0.54	-0.06	-0.08	-0.24	0.34	**1.00**															** **	** **
PHE	-0.36	-0.04	-0.30	0.00	0.13	0.05	0.51	-0.02	-0.38	-0.16	-0.08	0.00	0.15	0.15	0.34	0.04	-0.25	**0.85**	0.01	0.04	**0.58**	**0.64**	0.21	-0.37	**1.00**														** **	** **
BALA	-0.29	0.06	-0.23	-0.16	0.06	0.37	0.27	0.05	0.21	0.00	0.48	**0.78**	0.00	-0.24	0.43	-0.29	-0.18	-0.11	0.01	-0.17	0.21	0.23	-0.05	-0.19	-0.08	**1.00**													** **	** **
BABA	-0.29	0.28	-0.26	-0.14	**0.65**	-0.23	-0.07	0.41	-0.29	0.01	-0.07	0.05	0.34	-0.27	0.15	-0.17	-0.25	-0.12	0.15	-0.10	0.09	0.15	-0.15	-0.01	-0.16	-0.03	**1.00**												** **	** **
GABA	-0.46	0.28	-0.44	**0.55**	**0.81**	**-0.56**	0.30	0.18	-0.37	-0.31	0.01	0.27	0.51	-0.42	0.01	-0.36	-0.46	-0.13	0.14	**0.59**	0.05	0.21	**-0.57**	-0.37	0.10	0.16	0.21	**1.00**											** **	** **
HMCYS	-0.24	0.16	-0.30	-0.01	-0.22	0.15	**-0.54**	-0.28	0.34	**0.94**	-0.13	-0.11	0.01	-0.21	0.24	-0.19	-0.22	0.40	**0.71**	0.04	0.27	0.03	0.49	**0.72**	-0.05	-0.08	0.01	-0.40	**1.00**										** **	** **
ETH	-0.45	**0.63**	-0.43	-0.26	0.34	**-0.64**	0.00	**0.70**	**-0.64**	-0.19	0.07	0.39	**0.73**	**-0.64**	0.03	-0.35	**-0.52**	-0.03	0.17	-0.25	0.20	**0.56**	-0.49	-0.36	0.15	0.01	0.15	0.43	-0.32	**1.00**									** **	** **
TRP	-0.34	0.11	-0.36	-0.08	0.18	-0.19	-0.17	0.07	-0.04	0.22	-0.04	0.23	0.13	-0.23	0.48	-0.21	-0.33	**0.53**	0.20	0.04	**0.57**	0.40	**0.65**	0.01	0.34	0.09	0.20	-0.01	0.46	0.04	**1.00**								** **	** **
HYDLYS	-0.38	**0.62**	-0.34	-0.20	**0.58**	-0.35	0.16	0.18	-0.52	-0.14	0.01	0.48	0.44	-0.34	0.41	-0.15	-0.29	0.24	0.32	-0.11	0.49	**0.76**	-0.13	-0.20	0.35	0.28	0.10	**0.59**	-0.17	**0.62**	0.22	**1.00**							** **	** **
ORN	0.39	-0.42	0.42	-0.11	-0.34	0.74	0.49	-0.34	0.13	-0.25	-0.23	-0.26	**-0.66**	**0.91**	0.08	**0.77**	**0.54**	0.32	-0.48	-0.14	0.07	0.03	0.35	-0.14	0.34	-0.16	-0.22	-0.48	-0.11	**-0.53**	-0.18	-0.28	**1.00**						** **	** **
LYS	-0.34	0.06	-0.32	-0.26	0.00	0.21	0.40	-0.12	-0.10	0.04	0.16	0.16	0.04	0.06	**0.65**	0.12	-0.25	**0.85**	0.18	-0.22	**0.84**	**0.74**	0.40	-0.21	0.84	0.17	-0.20	-0.06	0.15	0.10	0.47	0.46	0.27	**1.00**					** **	** **
MHIS1	-0.41	**0.64**	-0.36	-0.21	**0.65**	-0.37	0.16	0.24	**-0.54**	-0.16	0.01	0.49	0.47	-0.36	0.42	-0.16	-0.32	0.21	0.31	-0.12	0.49	**0.76**	-0.14	-0.21	0.32	0.28	0.23	**0.61**	-0.18	**0.63**	0.25	**0.99**	-0.30	0.42	**1.00**				** **	** **
HIS	0.06	-0.39	0.04	-0.04	-0.32	0.51	0.30	-0.21	**0.64**	-0.04	**0.81**	-0.12	**-0.57**	0.24	**0.61**	0.14	0.07	0.15	-0.19	-0.13	0.24	-0.07	0.29	-0.21	0.15	0.25	-0.23	-0.31	-0.03	-0.30	0.12	-0.23	0.25	0.37	-0.25	**1.00**			** **	** **
CARNO	-0.06	-0.23	-0.11	-0.07	-0.19	-0.30	0.06	0.17	-0.14	-0.04	-0.05	-0.06	0.35	-0.27	-0.26	-0.16	-0.17	0.04	-0.09	-0.12	-0.07	0.02	-0.29	-0.14	-0.04	-0.22	-0.10	-0.01	-0.13	0.44	-0.15	-0.15	-0.21	-0.21	-0.16	-0.20	**1.00**		** **	** **
ARG	-0.32	0.31	-0.36	-0.17	-0.31	-0.03	-0.47	0.22	0.01	**0.68**	-0.21	-0.09	0.17	-0.26	0.09	-0.13	-0.35	0.41	0.46	-0.15	0.28	0.23	0.35	0.38	0.14	-0.27	-0.10	-0.46	**0.74**	0.14	0.43	-0.13	-0.07	0.20	-0.14	-0.09	0.21	**1.00**	** **	** **
NP	0.71	-0.56	0.75	-0.22	-0.46	0.73	0.27	-0.41	0.43	-0.37	0.22	-0.14	**-0.87**	**0.79**	0.04	0.55	**0.79**	-0.23	-0.64	-0.29	-0.26	-0.36	0.29	-0.05	-0.18	0.22	-0.29	-0.47	-0.32	-0.60	-0.38	-0.36	0.67	-0.06	-0.38	0.49	-0.27	-0.46	**1.00**	** **
P	**-0.71**	0.56	**-0.75**	0.22	0.46	**-0.73**	-0.27	0.41	-0.43	0.37	-0.22	0.14	**0.87**	**-0.79**	-0.04	-0.55	**-0.79**	0.23	0.64	0.29	0.26	0.36	-0.29	0.05	0.18	-0.22	0.29	0.47	0.32	0.60	0.38	0.36	**-0.67**	0.06	0.38	-0.49	0.27	0.46	-1.00	**1.00**

AAAA, α-amino adipic acid; AABA, α-aminobutyric acid; Ala, alanine; Arg, arginine; Asn, asparagine; Asp, aspartate; BABA, β-aminobutyric acid; BALA, β-alanine; Carno, carnosine, Cit, citrulline; Cys, cystine; Cysta, cystathionine; Eth, ethanolamine; GABA, γ-aminobutyric acid; Gln, glutamine; Glu, glutamate; Gly, glycine; His, histidine; Hom, homocysteine; Hpro, hydroxyproline; Hydlys, hydroxyl-lysine; Ile, isoleucine; Leu, leucine; Lys, lysine; Meh, 1-methyl histidine; Met, methionine; Orn, ornithine; Phe, phenylalanine; PHOS, phosphoethanolamine; Orn, ornithine, Pro, proline; Sar, sarcosine; SEP, phosphoserine; Ser, serine; Tau, taurine; Thr, threonine; Trp, Tryptophan; Tyr, tyrosine; Val, valine; NP, CLas-non permissive; P, CLas permissive

**Table 4:** Ratio of mean concentrations between *Candidatus* Liberibacter asiaticus (*C*Las)-permissive and non–permissive hosts for key amino acids identified as variables of most importance in partial least squares-discriminant analysis (PLS-DA).

Amino acid comparison	Category of CLas-host	Ratio of amino acid concentrations	Range of Ratio
Proline to Glycine	Permissive	24.7	6.3–45.2
	Non-Permissive	1.1	0–5.2
Proline to AABA	Permissive	232.4	97.2–3 82.1
	Non-Permissive	6.4	0–28.3
Proline to Threonine	Permissive	18.3	2.8–42.1
	Non-Permissive	0.4	0–1.4

## Discussion

In this study, the hypothesis that FAA contents of plant phloem sap and whole insect samples are strongly associated with *C*Las host status and could play a role in *C*Las growth and replication was evaluated. The results showed that FAA composition and relative concentrations greatly varied with host species, flush shoot growth stage and vector developmental stage. In all *Rutaceae* plants, young shoot phloem sap had higher numbers and concentrations of individual FAAs. In contrast, *D*. *citri* nymphs and adults contained the same numbers of individual FAAs and equivalent concentration per g of body weight.

Amino acids have been shown to play a key role in signaling between plants and pathogens in compatible interactions [29–36]. Strong variations in the FAA profiles of HLB-affected citrus tissue have also been reported [37–39]. Although changes in plant FAA profiles occur during plant-pathogen interactions, FAAs required for successful colonization must be present in adequate amounts in healthy plants prior to infection for successful pathogen growth. Concurrently, pathogen-inhibiting factors, including FAA composition must be absent or occur below toxic levels for successful host infection, colonization and establishment. An analysis of the *C*Las genome recently identified the presence of a tricarboxylic (TCA) cycle indicative of the utilization of a range of AAs as energy sources by *C*Las [14]. However, *C*Las is auxotrophic for a number of AAs including proline and must rely on its hosts as the primary sources for these AAs [14,40]. Thus, AA resources present in healthy hosts are a critical factor for successful *C*Las host colonization. In agreement with findings of the present study, healthy citrus varieties tolerant/resistant to HLB have been reported to contain higher levels of citrulline, glycine and ornithine [37,39] while proline, serine and aspartic acid were present in higher concentrations in most susceptible citrus varieties [37]. This has led to the hypothesis that variation in AA profiles between curry leaf, orange jasmine and citrus (‘Valencia’ sweet orange) may explain their differential responses to *D*. *citri* development and *C*Las growth [23].

Twelve FAAs are significantly related to CLas permissivity, of which seven were positively correlated to *C*Las non-permissive hosts and five were positively associated with *C*Las-permissive hosts. Notably, all of these amino acids had aspartate, glutamate or serine as precursors. Interestingly, AAs associated with *C*Las host susceptibility are either sulfur-containing AAs (methionine and taurine) or known to accumulate during stress (arginine and proline) or following injuries and mechanical damage (GABA), and are direct products of glutamate. The relative concentration of these five AAs did not significantly vary with flush shoot growth stage or *D*. *citri* developmental stage in *C*Las permissive hosts. In addition to their basic role as precursors for protein synthesis, many proteinogenic AAs such as arginine and proline have high nitrogen to carbon ratios and thus can serve as major storage and transport forms of organic nitrogen (especially during periods of stress) and subsequently metabolized for protein synthesis and energy production [32,41]. These AAs can potentially also serve as N and energy substrates for many microorganisms such as bacteria within plants [42]. Genomic analysis indicate that *C*Las is incapable of synthesizing arginine from glutamate [43] and may depend on its host for acquisition of this AA. Ethanolamine is used by many bacteria as a source of carbon and/or nitrogen [34], and is reported to foster the pathogenicity of many bacteria [44]. For instance, ethanolamine appears to be a key signal for initiation of virulence by *Escherichia coli* [45]. Sulfur (S) is a key constituent of many indispensable cell components and processes. In bacteria, S-containing AAs are the main pathway of S acquisition and play essential roles in their metabolism [46] including communication and regulation of virulence [47]. Interestingly, S-containing AAs were only present in *C*Las-permissive hosts and were not detected in non-permissive plants in agreement with a recent study [23]. This indicates that S-containing AAs may be either entirely absent or present below detection levels in *C*Las-non permissive plants. Nonetheless, such differential levels of S-containing AAs in the two *C*Las response categories evaluated in this study can contribute to determining their suitability for *C*Las growth.

In contrast to AAs that are positively related to *C*Las permissivity, the relative proportions of AAs positively correlated with *C*Las non-permissive plants (glycine, α-amino-butyric acid, phosphoethanolamine, threonine, serine, ornithine and citrulline) increased with flush maturity in all plant species. Serine and glycine are known to accumulate in plants in response to stress factors such as increased photorespiration or overexpression of proteases and peptidases [48,49]. Serine, derived from 3-phosphoglycerate is also a known precursor of glycine and the sulfur-containing AAs cysteine, cystathionine and methionine. HLB-affected citrus trees showed elevated concentrations of glycine, serine and threonine in phloem sap [37] and, of citrulline, glycine, ornithine and serine in leaf tissue [39], indicating possible roles for these AAs in citrus response to *C*Las infection. Furthermore, some of the AAs that are upregulated in HLB susceptible cultivars following *C*Las infection (e.g. citrulline and serine) were also naturally present at higher concentrations in the HLB-tolerant US-897 (*Citrus reticulata* ‘Cleopatra × *Poncirus trifoliata*) relative to HLB susceptible cultivars [39] corroborating the findings of this study.

Although many AAs were positively correlated either with *C*Las non-permissive or permissive hosts, only proline and glycine had PLS-VIP scores greater than the cut-off value of 1 using the 95% jackknife confidence interval. Hence, both AAs could be considered to be among the primary discriminating amino acids between *C*Las permissive and non-permissive hosts. Remarkably, proline and glycine are osmoprotectants produced in plants under osmotic stress conditions [43,50]. Proline, whose main biosynthetic pathway originates from glutamate as the precursor, is a well-known biomarker of water stress in plants since it accumulates to very high levels under drought conditions [51] and other stresses such as high salinity, heavy metal toxicity and high temperatures [52]. Proline is known to induce antioxidant defense gene expression in many organisms [53] and increasing evidence suggests that this AA plays an important role as a substrate for growth and respiration in bacteria. For *Helicobacter pylori*, proline is the preferred respiratory substrate during colonization of the human stomach [54]. In *E*. *coli*, proline increases oxidative stress tolerance [55]. Low-proline environments impair growth and *in vivo* survival of *Staphylococcus aureus* [56]. Many insects contained high levels of proline [57] that is a major fuel source for flight [58]. Therefore, entomopathogenic bacteria species are able to sense and exploit proline for expressing their virulence and initiating metabolism, thus identifying host niche [59].

Proline is the dominant FAA in phloem sap of *C*Las-permissive citrus ([20, 21], Fig 2) and in *D*. *citri* nymphs. Proline was also one of the two most abundant AAs found in periwinkle (along with aspartate) and in *D*. *citri* adults (along with glutamine) in this study (Fig 2). It is conceivable that high proline contents in permissive hosts may play a key role as an activator of *C*Las secondary metabolite virulence factors, but also as an energy source to sustain the pathogen as it establishes, multiplies and spreads. In contrast, very low concentrations of proline (<3%) were detected in *C*Las non-permissive plants (Fig 2) with the exception of young orange jasmine shoots that contained 16% of proline in their phloem sap. This may explain the unsuitability of these plant species for sustainable *C*Las establishment and growth.

Orange jasmine has been reported to be a possible host of *C*Las but considerable variability exists in its infection rates, bacterial titer and persistence due to an apparent lack of *C*Las fitness in the plant phloem [60]. Lower *C*Las titer levels have also been reported in orange jasmine and orange jasmine-reared psyllids compared to citrus and citrus-reared psyllid, respectively [61]. It is therefore plausible that *C*Las successfully colonizes phloem sap of young orange jasmine shoots, but does not persist in the phloem as the flush shoots mature due to rapid metabolic shifts resulting in altered AA profiles, notably a drastic reduction in the proportion of proline, as the shoot matures. Genomic analysis indicate that *C*Las uses proline as a growth factor [40,62]. Based on reports of increased proline concentrations in HLB-affected trees [39,63], and strong correlations between high proline levels in plant tissue and HLB susceptibility in citrus, Cevallos-Cevalloset al. [37] hypothesized that proline abundance facilitates *C*Las survival and spread *in planta*. Taken together, these results point to the critical role played by proline in *C*Las host recognition, successful colonization and growth. Paradoxically, as proline level increased in plants in response to stress [64] and to *C*Las infection [39], *C*Las adaption to the proline-rich environment will also favor post-infection survival/multiplication, thus leading to higher bacterial titer in HLB-affected plants. This pathogen-host dynamic demonstrates how biotrophic organisms exploit their hosts for growth, multiplication and survival.

Glycine is the simplest known AA and, in its methylated form as glycine betaine, is another important osmolyte that adjusts osmotic balance in bacteria, animals, and angiosperms as a common response for host protection against environmental stresses such as salt, drought, and extreme temperatures [50,65]. Exogenous applications of glycine have been shown to increase plant tolerance to abiotic stress [50,66]. Although glycine is used as a metabolic product in some bacteria, a high concentration of glycine is known to have toxic effects that inhibit growth in many bacteria [31]. Glycine is known to inhibit the synthesis of a peptidoglycan component of the bacterial cell wall [67]. As the bacterial cell wall is thinner in gram-negative than in gram-positive bacteria, it is thought that the amount of glycine required to suppress gram-negative bacterial proliferation is lower than that required to suppress proliferation of gram-positive bacteria [31]. Due to its low mammalian toxicity, glycine has been used as an antibacterial agent in foods. Hence, as *C*Las is a gram-negative bacterium, it is likely that high concentration of glycine in phloem sap of plants can interfere with its metabolism. The percentage of glycine in the AA pool of *C*Las non-permissive plants was 2.3 to 8-fold higher than that in their *C*Las-susceptible counterparts in agreement with a previous report [37].

As both *C*Las promoting and inhabiting AAs occur simultaneously in phloem sap, it is very likely that the outcome of host-*C*Las interaction will depend on ratios of these two types of AAs. The outcome of this study suggests that the proline-to-glycine ratio may be a critical discriminating factor for host permissivity to *C*Las, with higher and stable values (6 to 45) characterizing permissive hosts and lower values (<0.6) being emblematic of non-permissive hosts. Using this criterion, orange jasmine could be classified as a transient host of *C*Las with the phloem sap of its young shoots having a proline-to-glycine ratio of 5.2 whereas its mature shoots had a mean ratio of 0.3.

In conclusion, this study indicates the suitability of FAA profiling for *C*Las host response discrimination. Although the dimension reduction techniques resulted in clear discrimination of hosts into two groups based on their *C*Las permissivity and gave a clear lead to AAs correlated with either *C*Las-permissive or non-permissive hosts, identification of their roles in *C*Las growth and HLB development in hosts will require additional biological studies. As efforts to identify and develop HLB tolerant/resistant cultivars expand, metabolite profiles such as the proline-to-glycine ratios identified in this study could be exploited as biomarkers for rapid screening of parental and progeny citrus genotypes in resistance breeding programs.

## Supporting information

S1 Table Report for Venn diagram indicating shared and unique free amino acids (FAA) in whole *D*. *citri* nymphs and adults and phloem sap of young flush shoots of different plants tested. (PDF)

S2 Table Report for Venn diagram indicating shared and unique free amino acids (FAA) in whole *D*. *citri* nymphs and adults and phloem sap of mature flush shoots of different plants tested. (PDF)

S1 Fig **A)** VIP scores: Amino acids whose relative concentrations are involved in *C*Las host discrimination ordered by index score of variable importance on the Protection greater than 1 (VIP-1) criterion.
**B)** Orthogonal projection coefficients for the comparison between permissive and non-permissive hosts of *C*Las. Negative values represent FAA positively correlated with CLas-permissive hosts whereas negative values correspond to those with higher concentrations in non-permissive plants.
(TIF)

S1 Dataset Amino acid analysis CLas-permissive and non-permissive hosts. (XLS)
