# Supplementary material for: Contrasting amino acid profiles among permissive and non-permissive hosts of Candidatus Liberibacter asiaticus, putative causal agent of Huanglongbing
Source: PLoS One. 2017 Dec 13;12(12):e0187921. doi: 10.1371/journal.pone.0187921 (PMC5728503; doi:10.1371/journal.pone.0187921)
Supplement: S1 Table — (PDF) [file pone.0187921.s001.pdf]

S1 Table: Report for Venn diagram indicating shared and unique free amino acids (FAA) in whole *D. citri* nymphs and adults and phloem sap of young flush shoots of different plants tested.

**List names**	**Number of unique FAAs detected**	**Number of common or unique FAAs present in samples**	
**Overall number of unique elements**	**38**	**Number**	**Overall number of unique elements**
D. citri adults	31	18	Aspartate, Lysine, histidine, asparagine, glycine, proline, ornithine, phenylalanine, valine, threonine, glutamate, ornithine, serine, phosphoserine, alanine, arginine, phosphoethanolamine, α-amino-butyric acid, γ-amino-butyric acid
D. citri nymphs	30		
Curry leaf	23		
Grapefruit	35		
Lemon	35		
Orange jasmine	25		
Periwinkle	22		
Sweet orange	35		
White sapote	28		
D. citri adults, *D. citri* nymphs, Curry leaf, Grapefruit, Lemon, Orange jasmine, Sweet orange, White sapote,		4	Leucine, α-amino-adipic acid, Tyrosine, Isoleucine
D. citri adults, *D. citri* nymphs, Curry leaf, Lemon, Orange jasmine, Periwinkle, Sweet orange, White sapote,		1	Glutamine
D. citri adults, *D. citri* nymphs, Grapefruit, Lemon, Orange jasmine, Sweet orange, White sapote		2	β-alanine, sarcosine
D. citri adults, *D. citri* nymphs, Grapefruit, Lemon, Periwinkle, Sweet orange, White sapote		1	Methionine
D. citri adults, *D. citri* nymphs, Grapefruit, Lemon, Periwinkle, Sweet orange		1	Cystine
Grapefruit, Lemon, Sweet orange, White sapote		1	Ethanolamine
Grapefruit, Lemon, Periwinkle, Sweet orange		1	Hydroxyproline
D. citri nymphs, Grapefruit, Lemon, Sweet orange		1	Tryptophan
D. citri adults, Grapefruit, Lemon, Sweet orange		1	Taurine
*D. citri a*dults, *D. citri* nymphs, White sapote		1	Cystathionine
Grapefruit, Lemon, Sweet orange		3	Hydroxylysine, carnosine, 1-methyl histidine
*D. citri a*dults, Grapefruit, Lemon		1	β-amino-butyric acid
Curry leaf, Sweet orange		1	Citrulline
*D. citri a*dults, *D. citri* nymphs		1	Homocysteine
