# Supplementary material for: Contrasting amino acid profiles among permissive and non-permissive hosts of Candidatus Liberibacter asiaticus, putative causal agent of Huanglongbing
Source: PLoS One. 2017 Dec 13;12(12):e0187921. doi: 10.1371/journal.pone.0187921 (PMC5728503; doi:10.1371/journal.pone.0187921)
Supplement: S2 Table — (PDF) [file pone.0187921.s002.pdf]

S 2 Table: Report for Venn diagram indicating shared and unique free amino acids (FAA) in whole *D. citri* nymphs and adults and phloem sap of mature flush shoots of different plants tested.

**List names**	**Number of unique FAAs detected**	**Number of common or unique FAAs present in samples**	
**Overall number of unique elements**	**36**	**Number**	**Overall number of unique elements**
D. citri adults	31	15	Aspartate, Lysine, histidine, glycine, phenylalanine, valine, threonine, glutamate, serine, phosphoserine, alanine, arginine, phosphoethanolamine, α-amino-butyric acid, γ-amino-butyric acid
D. citri nymphs	30		
Curry leaf	22		
Grapefruit	26		
Lemon	27		
Orange jasmine	21		
Periwinkle	22		
Sweet orange	29		
White sapote	19		
D. citri adults, *D. citri* nymphs, Curry leaf, Grapefruit, Lemon, Orange jasmine, Sweet orange, White sapote,		1	Tyrosine
D. citri adults, *D. citri* nymphs, Curry leaf, Lemon, Orange jasmine, Periwinkle, Sweet orange, White sapote,		1	Ornithine
D. citri adults, *D. citri* nymphs, Curry leaf, Grapefruit, Lemon, Orange jasmine, Periwinkle, Sweet orange		2	Asparagine, proline
D. citri adults, *D. citri* nymphs, Curry leaf, Grapefruit, Lemon, Sweet orange		2	Leucine, sarcosine
D. citri adults, *D. citri* nymphs, Grapefruit, Lemon, Sweet orange		2	Glutamine, methionine
D. citri adults, *D. citri* nymphs, Grapefruit, Lemon, Sweet orange		1	α-amino-adipic acid
D. citri adults, *D. citri* nymphs, Orange jasmine, Sweet orange		1	Cystathionine
*D. citri a*dults, *D. citri* nymphs, Lemon, Sweet orange		1	Isoleucine
Orange jasmine, Periwinkle, White sapote		1	Hydroxyproline
D. citri nymphs, Lemon, sweet orange		2	Carnosine, ethanolamine
*D. citri a*dults, *D. citri* nymphs, Periwinkle		1	Cystine
D. citri adults, Grapefruit		1	Taurine
*D. citri a*dults, Sweet orange		1	β-amino-butyric acid
*D. citri a*dults, *D. citri* nymphs		3	β-alanine, homocysteine
D. citri nymphs		1	Tryptophan
