# Supplementary figures and images for: Contrasting amino acid profiles among permissive and non-permissive hosts of Candidatus Liberibacter asiaticus, putative causal agent of Huanglongbing
Source: PLoS One. 2017 Dec 13;12(12):e0187921. doi: 10.1371/journal.pone.0187921 (PMC5728503; doi:10.1371/journal.pone.0187921)

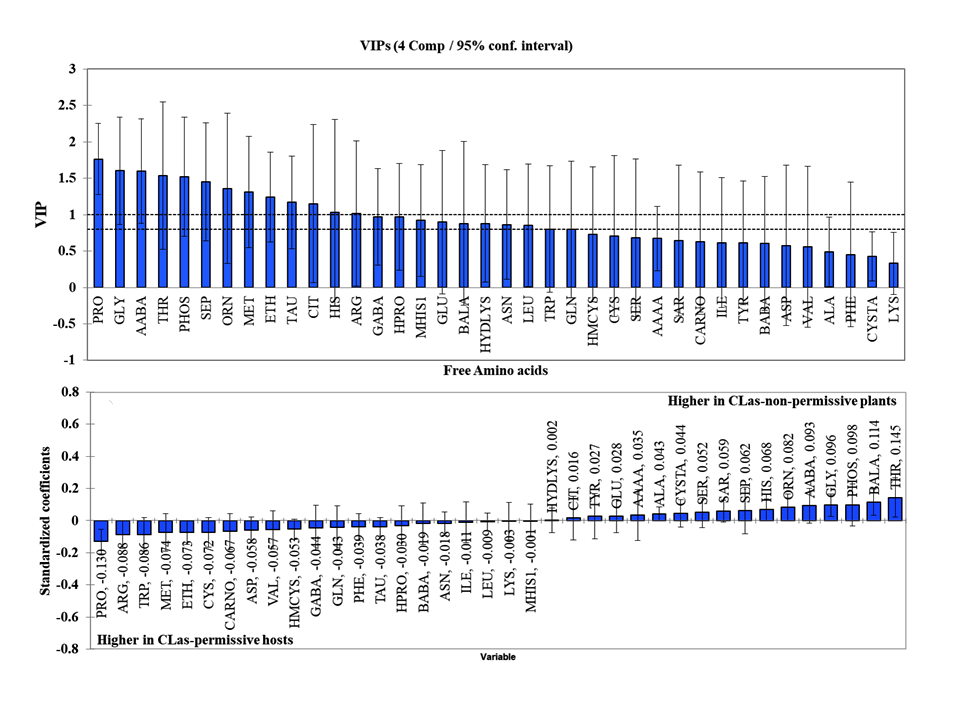

Supplement: S1 Fig — A) VIP scores: Amino acids whose relative concentrations are involved in CLas host discrimination ordered by index score of variable importance on the Protection greater than 1 (VIP-1) criterion. B) Orthogonal projection coefficients for the comparison between permissive and non-permissive hosts of CLas. Negative values represent FAA positively correlated with CLas-permissive hosts whereas negative values correspond to those with higher concentrations in non-permissive plants. (TIF) [file pone.0187921.s003.tif]
